# An Information-Based Approach for Mediation Analysis on High-Dimensional Metagenomic Data

**DOI:** 10.3389/fgene.2020.00148

**Published:** 2020-03-13

**Authors:** Kyle M. Carter, Meng Lu, Hongmei Jiang, Lingling An

**Affiliations:** ^1^ Interdiciplanary Program in Statistics and Data Science, The University of Arizona, Tucson, AZ, United States; ^2^ Department of Statistics, Northwestern University, Evanston, IL, United States; ^3^ Department of Epidemiology and Biostatistics, The University of Arizona, Tucson, AZ, United States; ^4^ Department of Biosystems Engineering, The University of Arizona, Tucson, AZ, United States

**Keywords:** high-dimension, mediation analysis, information, nonparametric, microbiome, host genome

## Abstract

The human microbiome plays a critical role in the development of gut-related illnesses such as inflammatory bowel disease and clinical pouchitis. A mediation model can be used to describe the interaction between host gene expression, the gut microbiome, and clinical/health situation (e.g., diseased or not, inflammation level) and may provide insights into underlying disease mechanisms. Current mediation regression methodology cannot adequately model high-dimensional exposures and mediators or mixed data types. Additionally, regression based mediation models require some assumptions for the model parameters, and the relationships are usually assumed to be linear and additive. With the microbiome being the mediators, these assumptions are violated. We propose two novel nonparametric procedures utilizing information theory to detect significant mediation effects with high-dimensional exposures and mediators and varying data types while avoiding standard regression assumptions. Compared with available methods through comprehensive simulation studies, the proposed method shows higher power and lower error. The innovative method is applied to clinical pouchitis data as well and interesting results are obtained.

## Introduction

Humans maintain a close symbiotic relationship with trillions of microorganisms that live upon and within their bodies. The human body relies on assorted communities of microbes to develop bodily functions such as metabolism and immune response as well as to protect the body from infections from harmful pathogens. Researchers have begun to recognize the importance of the interactions between host and microbiota and how they may impact human health. In particular, studying this interaction has become a key topic in numerous fields of research such as immunology (Rogers and Wesselingh, 2016; Rooks and Garret, 2016), oncology (Taur and Parmer, 2016), and metabolomics (Rostami et al., 2015; Galla et al., 2017; Kurilshikov et al., 2017). The current Integrative Human Microbiome Project (IHMP) aims to record behavior over time for host biology and the metagenome for the onset of Inflammatory Bowel Disease and Type 2 Diabetes as well as for neonatal development. With progressively more data available, a growing research interest has emerged for integrative analysis of multiple omics data, for example, host transcriptome and human microbiome data.

One popular approach for integrating multiple omics datasets is mediation analysis. A mediation model aims to extract the mechanisms by which an exposure impacts the outcome variable by considering a set of potential variables which may mediate the effect. Identifying these mechanisms is a vital step in developing effective medication and therapy as well. In particular, the microbial community could be easier to manipulate using antibiotics and probiotics.

Simple mediation models with only one exposure and one mediator have been widely used in psychology for several decades (MacKinnon et al., 2006; Agler and De Boeck, 2017), with most recent notable development focused on models with multiple mediator variables (Daniel et al., 2015). However, the application of mediation models for biological data has introduced additional challenges, including the difficulty of incorporating multiple, high dimensional omics datasets with varying data structures. In this research, we aim to develop a nonparametric framework for mediation analysis to avoid the assumptions and pitfalls of current mediation models.

## Materials and Methods

### Background

**Figure 1:**
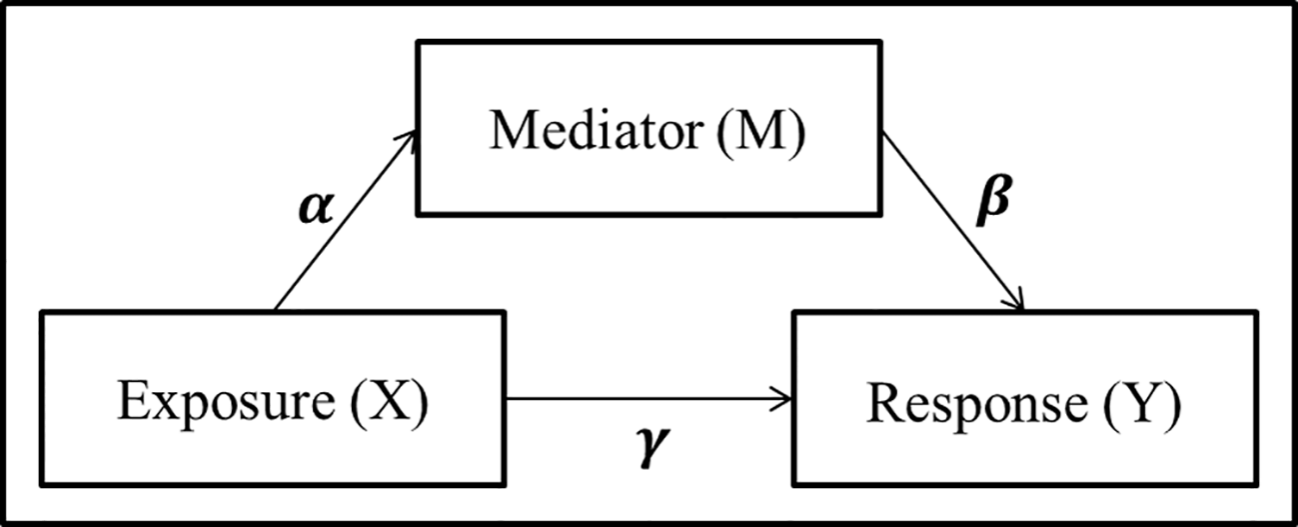
Panel model representation of a mediation model with a single exposure *X*, single mediator *M*, and single response *Y*, and the associated coefficients used for linear structural equation modeling.

A simple mediation model aims to explain the mechanisms that underlay the relationship between an exposure variable (X) and a response variable (Y), by considering a tertiary mediator variable (M) which may mediate the effect of the exposure on the response (**Figure 1**). The total effect of the exposure variable can be decomposed into the *direct effect*, effect from exposure to response directly, and the *indirect effect*, effect of the exposure which is mediated by the mediator variable.

A mediation model is most commonly examined parametrically utilizing a linear structural equation model (LSEM):

(1) Y=γ′X+ε

(2) $$M=\alpha X+\varepsilon_{M}$$

$$Y=\gamma X+\beta M+\varepsilon_{Y}$$

(3) $$=(\gamma +\alpha \beta )X+\beta \varepsilon_{M}+\varepsilon_{Y}$$

where *γ′* and *γ* represent the total effect and direct effect, respectively. Baron and Kenny (1986) proposed to detect whether an indirect effect exist by testing either the product *αβ* = 0 or the difference between the total and direct effects *γ′* – *γ* = 0. In addition to the traditional mediation assumptions of causal direction (i.e., additive effects and no unmeasured confounders or sequential confounders) (MacKinnon et al., 2006; Vanderwheele and Vansteelandt, 2014; Preacher, 2015), the LSEM approach requires standard regression assumptions such as linearity, no collinearity, known link function, exponential distribution of the error term, and sample size larger than parameter space. While the LSEM structure has seen widespread use and success in psychology applications where mediation analysis includes a single mediator and continuous exposure variables, many of these assumptions are violated in the context of genomics and metagenomics studies with counts data.

In response to these challenges, new statistical methods have been developed in the last few decades in an attempt to apply mediation modeling approaches for neural and biological data. Boca et al. (2014) constructed a distribution of the correlation between parameters by permuting the outcome in each of the LSEM equations. Huang and Pan (2015) developed a Monte-Carlo procedure to evaluate the mediation effect of high-dimentional continuous mediators. Huang et al. (2015) performed an omnibus test by comparing L_1_ normalized terms from three logistic regression models based on the structural equations model. Kim et al. (2016) and Nguyen et al. (2016) utilized binary exposure to generate natural direct and indirect effect measures *via* expectation differences. Zhang et al. (2016) used minimax concave penalty regularized logistic regression models to estimate *β* effect (in eq(3)). Recently, Sohn and Li (2019) proposed a causal composition mediation model (CCMM) specifically for microbiome mediators which utilized a bootstrap covariance matrix to perform log-contrast compositional regression. While these approaches may avoid concerns associated with the n< < p paradigm (i.e., sample size is smaller than the parameter space), they often require a single exposure variable and a linear relationship between parameters. Many additionally enforce certain data type such as binary exposures or continuous responses.

In this research, we aim to evaluate the presence of indirect effects by developing a nonparametric framework based on information transfer. While applications of information theory in a biological context have been seldom, it has achieved some success in feature selection for gene expression data (Meyer et al., 2008; Radovic et al., 2017). Recent advances in this field include alternatives for finding relative contribution of variables using entropy methods. Radovic et al. (2017) approached this problem by introducing a penalty term for mutual information shared between selected variables. Liu et al. (2016) assigned a measure of feature quality by comparing conditional information of a variable on an outcome conditioned upon k-nearest-neighbor variables. By utilizing information-based methods, in our research, there is no need to assume underlying distributions or data types of genomic/metagenomic data, or response variable (e.g., clinical outcome) while nonlinear or non-additive relationships between variables can be explored.

### Methods

Recent research has discovered that the abundance and diversity of the microbiome have an impact on the expression of human genes (Blekhman et al., 2015; Bonder et al., 2016; Davenport, 2017). In this study, we will focus on treating microbes as mediators for host genes. However, the proposed method itself is very general and can be applied in other types of studies, e.g., genomic or epigenomic study, or even studies in other fields.

To discover which microbial taxa mediate the effect of gene expression on a clinical outcome, we propose a nonparametric framework based on information theory feature reduction techniques, termed as Nonparametric Entropy Mediation (NPEM). Information theory compares joint distributions of two or more variables with the marginal distributions of subsets to measure association between variables. This can capture nonlinear and non-additive associations by observing changes in distribution of the outcome as compared to distance based and regression modeling approaches which can only capture linear association with the outcome (Roulston, 1999). The information can be measured using Shannon Entropy and Mutual Information (MI) (Shannon, 1949). Shannon entropy represents the uncertainty, potential information, from a discrete random variable or random vector, and is defined as amount of information produced by a stochastic process:

(4) $$H(X)=-\Sigma_{x\in X}p(x)\log p(x),$$

where *p*(*x*) represents the probability of observing *X* = *x* (if the variable is continuous, this definition is redefined by using the integral across the domain for continuous density functions instead of the summation across the domain of events). Shannon entropy of a multivariate process between two variables X and Y can be calculated using joint Shannon entropy:

(5) $$H(X,Y)=-\Sigma_{x\in X}\Sigma_{y\in Y}p(x,y)\log p(x,y),$$

where *p*(*x*,*y*) represents the probability of observing *X* = *x* and *Y* = *y* (note: the notations *X* and *Y* here are just two common variables, different from the notations in the LSEM in *Background*).

Mutual information (MI) is defined as the overlap of information produced by multiple stochastic processes:

MI(X, Y)=H(Y)+H(X)−H(X,Y)

(6) $$=\Sigma_{x\in X}\Sigma_{y\in Y}p(x,y)\log\frac{p(x,y)}{p(x)p(y)}.$$

Mutual information can be used as a measure of dependency between the variables in a multivariate stochastic process. If the included variables are independent, the information metric is zero.

To capture the unique mutual information from a variable *X*, we additionally define the *contributed information* to be the mutual information of one variable given a set of measured variables (***W***):

(7) $$C(X,Y,W)=MI(X,Y)-\Sigma_{w\in W}\frac{MI(X,w)}{{\left\|W\right\|}^{2}}$$

**Figure 2:**
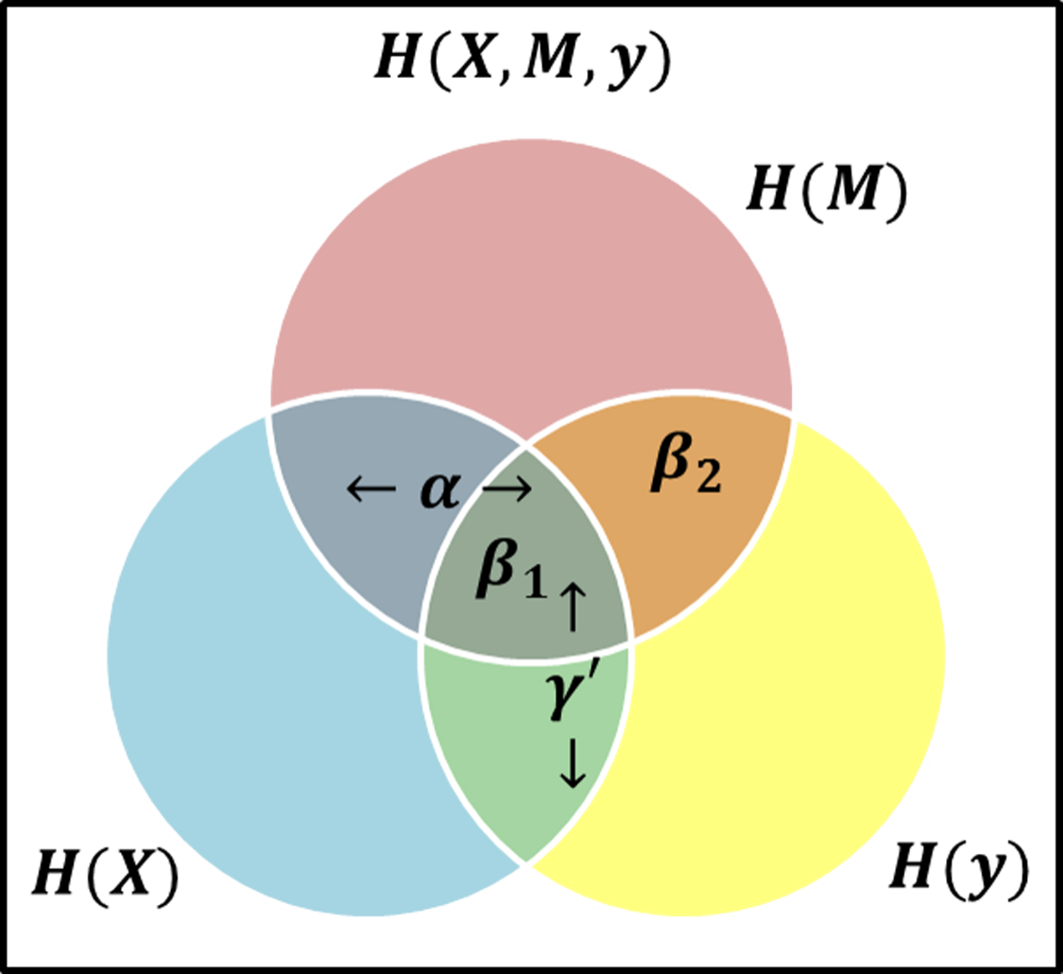
Venn Diagram visual representation of information content and the areas representative of model effects, α represents the relationship (i.e., intersection) between the exposure (blue circle) and mediator (red circle), *β_1_* is the relationship between all three variables, i.e., at the intersection of three circles, while *β_2_* represents the relationship between the mediator and response (yellow circle) excluding the exposure, i.e., the area of intersection of red and yellow circles, but not in blue, and *γ′* is the overlap of blue and yellow circles, representing the total effect.

To investigate the mediated relationship between host gene expression and a clinical outcome, we propose to construct the mediation model as a multivariate stochastic process generating the set of *I* genes (***X*** = {*X*_1_,…,*X*_I_}), the set of *J* microbial taxa (***M*** = {*M*_1_,…,*M_J_*}), and a clinical outcome *Y* (throughout the text of this paper we use bold symbols to represent sets of variables). If we maintain the causal direction and no intermediary confounding assumptions, we can examine the relationship between variables using the mutual information between variables from the stochastic processes. To mimic current LSEM structure, we define *γ′* as a label of relationship between ***X*** and *Y, α* as the relationship between ***X*** and ***M***, and *β* as the relationship between ***M*** and *Y* when ***X*** is also included. Thus, we use these labels to represent the relationships between the variables based on the theory information in **Figure 2**.

Consider the *β* effect from ***M*** to *Y* as the overlap in information contained by ***M*** and *Y*, then it can be decomposed into *β_1_* representing the overlap of *α* and *β*, and *β_2_* representing the unique information from ***M*** as shown in **Figure 2** such that *β* = *β_1_* + *β_2_*. Note that *β_2_* represents the value *βϵ* *_M_* in equation (3). If *β_2_* ≠ 0, then it follows *β* ≠ 0. Consider two possible outcomes when *β_2_* = 0: 1) if *β_1_* = 0 and *β_2_* = 0, then ***M*** does not offer any information about *Y* and there is no mediation effect. This is equivalent to *β* = 0 and by extension *αβ* = 0 in the LSEM framework; 2) if *β*_*1*_ ≠ 0 and *β_2_* = 0, all information ***M*** provides about *Y* is also contained in ***X***. Due to perfect collinearity, no conclusion can be drawn about the existence of mediation effects. For the purposes of our study, we will consider this scenario as not a mediation effect. Thus, the overlap of all variables is not sufficient and any scenario where *β_2_* = 0 would not be considered a mediation effect. The existence of mediation effects can be captured by measuring *α* and *β_2_*. The two relationships *α* and *β_2_* as shown in **Figure 2** can be expressed in terms of mutual information as *MI*(***X, M***), and *MI*(***M**, Y* ) respectively.

In order to capture the effect of each gene or each taxon individually, we additionally consider collinearity between the variables. We will use contributed information to measure the relationship between gene *i* and taxon *j*, α*_i,j_*, as *C*(*X_i_*,*M_j_*,***S***), and the relationship between taxon *j* and the response (for the purpose of explanation we use one clinical response variable) *Y*, *β_2_*, as *C*(*M_j_*,*Y*,***T***), where ***S*** and ***T*** represent a subset of other genes and other microbial taxa, respectively.

To non-parametrically estimate the mutual information and contributed information metrics, we employ kernel density estimation to approximate the distribution of each variable or a set of variables. To allow for varying data types in a joint distribution, we employ kernel product estimation developed by Li and Racine (2003). The choice of kernel will depend on the structure of the data. For continuous data, the distribution will be approximated using a second order Gaussian kernel, which is a common choice due to its smoothness and an ideal choice when integration is required. Distributions of discrete data will be approximated using an Aitchison-Aitken kernel to handle discrete entry frequencies. To avoid overfitting, bandwidths for kernels are approximated using Silverman’s Rule of Thumb (Silverman, 1986). To get an accurate density estimator we only need to know the data type but not the shape.

In high resolution sequencing studies, limited genetic material and PCR amplification biases can lead to many OTUs (operational taxonomic units) with zero count, even when those taxa exist within a subject’s gut microbiota. However, a concentration of counts at zero can lead to a problem when estimating the distribution using a Gaussian kernel density estimator. Most notably, the decreased variance can lead to smaller estimates for the kernel bandwidth. We propose two approaches for mediation testing using mutual information. In the simplest case, we use a single Guassian kernel to estimate the distribution of OTU abundance and to calculate the contributed information. We refer to this single kernel approach as a univariate entropy measure. To better represent the microbiome data and to avoid some of the potential pitfalls of kernel density estimation, we propose a bivariate approach which decomposes the microbiome data into two parts: presence-absence represented by an Aithison-Aitken kernel and nonzero counts represented by a Gaussian kernel. Contributed information metrics can be calculated separately for both presence–absence and nonzero counts, providing two measurements for each mediator. We refer to this two-kernel approach as a bivariate entropy measure.

#### Univariate Entropy Measure

When calculating mutual information, theoretically, the information metric should be zero if the variables are independent; however finite sample sizes and bandwidth approximation for the kernel density estimates may lead to a bias in the observed information. Out of a large number of taxa in a study, generally only some of them play mediating effect. Under this very general assumption, a vast majority of the signals observed are due to this bias effect. Therefore, we can search for information metrics which are substantially higher than the expected bias, as this indicates a true relationship between variables. For a particular taxon (*j)* to be a mediating taxon, there must be significant relationships from at least one gene through it to the response. Just like the regression model in Eq (2) where all exposure variables ***X*** are included for each mediator variable *M_j_*, α*_i,j_* (representing the relationship between the exposure variable *X_i_* and mediator *M_j_*) must be evaluated across all exposures simultaneously within each fixed taxon *j*. For each taxon *j* the hypotheses are:

$$H_{0}:C(X_{i},M_{j},S)\leq \varphi_{\alpha ,j},\forall i\in \{1,\ldots ,I\}\text{ OR }C(M_{j},Y,T)\leq \varphi_{\beta 2}$$

$$Ha:\exists i\in \{1,\ldots ,I\}:C(X_{i},M_{j},S)>\varphi_{\alpha ,j}\;\;\&\;C(M_{j},Y,T)>\varphi_{\beta 2}$$

The parameters *φ_α,j_* and *φ_β_*_2_ represent the expected bias for contributed information with a fixed taxon *j* and *Y* respectively. Since the mutual information score should be zero for independent random variables, the bias terms *φ_α,j_* and *φ_β_***_2_** are conservatively estimated as the mean contributed information scores for taxon *j* and currently unselected genes as defined below, respectively:

(8) $$\varphi_{\alpha ,j}=\Sigma_{X_{i}\in (X-S)}\frac{C(X_{i},M_{j},S)}{\left\|(X-S)\right\|}$$

(9) $$\varphi_{\beta 2}=\Sigma_{M_{j}\in (M-T)}\frac{C(M_{j},Y,T)}{\left\|(M-T)\right\|}$$

where ***X*-*S*** represents the set of genes which are currently unselected and ***M*-*T*** represents the set of OTUs which are currently unselected. For our definition, both the contributed information and the expected bias depend on the components of set ***S*** or ***T***. We propose to iteratively select the best predictive genes or taxa based on their contributed information and update ***S*** or ***T*** respectively after each selection by using a greedy search algorithm. Under this paradigm, we compare the largest contributed information to the average contributed information as defined in equations (8) and (9). This lends itself naturally to outlier detection tests which compare the maximum value to the mean for potential outlier points. Since there could be multiple features which contain true contributed information signals, we opt to use an iterative one-sided Extreme Studentized Deviate (ESD) test (Grubbs, 1950), which was developed for unusually high value detection. We evaluate a series of G statistics (Grubbs, 1950) as follows:

$$G=\frac{C_{\left(1\right)}\left(\ldots \right)-\bar{C\left(\ldots \right)}}{sd\left(C\left(\ldots \right)\right)}$$

where *C*_(1)_ represents the highest contributed information to be compared, either for the relation between taxon (**j**) and genes, or for the relation between the outcome and taxa. $\bar{C\left(\ldots \right)}$ stands for the average of contributed information and sd represents standard deviation. Under the null hypothesis, the *G* statistic follows a central *t*-distribution with degrees of freedom *df*-2, where *df* represents the number of remaining unselected features. However, since the contributed information could change at each step, there is still uncertainty on when the hypothesis test should be performed. We propose **Algorithm 1** which performs the hypothesis test at each iteration of the greedy search algorithm (NPEM : UV). To be specific, at each step of the algorithm, the contributed information from each gene to a fixed taxon or from each taxon to the clinical outcome is re-evaluated to identify the most informative feature. The highest value of contributed information is recorded, the hypothesis test is performed, and the selected feature is removed from the set of explanatory variables and added to the set of priors ***S*** or ***T***. A modified version which performs the hypothesis test after the completion of the greedy search is provided in **Supplementary File** as **Algorithm 1**′ (NPEM : UVS). The details and trade-offs of each algorithm are elaborated in the **Supplementary File**.

**Algorithm 1:** Non-Parametric Entropy Mediation: Univariate Test (NPEM:UV).

Input: ***A*** = {*A*_1_,*A*_2_,…,*A_K_*}: Set of explanatory variables; B: Response variable
1. Initialize an empty set ***W***.
2. Evaluate Contributed Information *C_i_* = *C*(*A_i_*,*B*,***W***) for each *A_i_* which is not in ***W***. When ***W*** is empty, *C_i_* = *MI*(*A_i_*,*B*).
3. Let ***C*** denote the vector of the *C_i_* values, and *C*_(1)_ denote the largest Contributed Information.
4. Calculate Grubb’s ESD Test Statistic: $G=\frac{C_{\left(1\right)}-\bar{C}}{sd\left(C\right)}$, where $\bar{C}$ is the average value and sd represents standard deviation.
5. Perform significance test with the distribution *t_df_*_-2_ to obtain p-value, where *df* is the length of ***C***.
6. If the p-value is below a threshold (e.g., 0.05), move the variable *A*_(1)_ corresponding to the largest value *C*_(1)_ into set ***W***.
7. Repeat steps 2 through 6 until a specified threshold (e.g. 0.05) is reached or until two or fewer variables remain.
8. For the variables which do not belong to ***W***, assign the p-value to be 1.
9. For each response variable, apply FDR correction (Benjamini and Hochberg, 1995) to the p-values of all explanatory variables.

This algorithm is general and can be applied to evaluate the significance of all *α* and *β_2_* relationships defined in Methods. For the *α* relationship, ***A*** is the full gene set ***X*** and *B* is an individual microbial taxon (*M_j_*), and the resulting p-value *p_α,__j_* is the FDR corrected p-values. For the *β_2_* relationships, ***A*** is the set of all microbial taxa ***M*** and *B* is the clinical response (*Y*). The resulting p-value *p_β,__j_* is FDR corrected. To complete the hypothesis test for mediation effects, we composite the results with conservative measure *p_j_* = max (*p_α,__j_,p_β,__j_*), which represents the final p-value for testing the mediation effect of taxon *j*.

#### Bivariate Entropy Measure

When we represent the abundance of each microbial taxon by decomposing the feature into presence-absence and nonzero counts, the contributed information can be calculated for both presence-absence and nonzero counts individually. Our final decision will leverage both contributed information scores. To test whether a relationship is significant or not, we propose a general hypothesis as follows:

$$H_{0}:\|\vec{C}\|\leq \varphi vs.H_{a}:\|\vec{C}\|>\varphi$$

where $\left\|\vec{C}\right\|$ represents any norm or distance metric for the vector of two contributed information metrics $\vec{C}$ from zero and nonzero counts. To account for the difference in scale and correlation between presence-absence and nonzero counts, we will utilize Mahalanobis distance (Mahalanbois, 1936):

$$MD(\vec{C})=\sqrt{(\vec{C}-\vec{\mu })\prime \Sigma^{-1}(\vec{C}-\vec{\mu }),}$$

where $\vec{\mu }$ represents the vector of means for $\vec{C}$ and Σ represents the covariance of the two contributed information scores in $\vec{C}$. The Mahalanobis distance is distance metric which projects data along its principal components. Each axis is re-scaled to ensure a mean value of zero and variance of 1. By projecting the two contributed information scores onto their principal components, we no longer need to consider correlation between scores. We can now rewrite our hypothesis using the distance from expected bias:

$$H_{0}:MD(\vec{C})\leq \varphi \;\text{vs.}\;H_{a}:MD(\vec{C})>\varphi$$

As in the univariate case (i.e., do not separate the zero and nonzeros counts for each taxon) in *Univariate Entropy Measure*, for a particular taxon to be a mediating taxon, there must be a significant mediation structure or bridge from at least one gene and then through the taxon to the clinical response. For each fixed taxon j, the hypotheses are as follows:

$$H_{0}:MD\left(\vec{C_{\alpha ,i,j}}\right)\leq \varphi_{\alpha ,j},\forall i\in \left\{1,\ldots ,I\right\}\;OR\;MD\left(\vec{C_{\beta_{2},j}}\right)\leq \varphi_{\beta_{2}}$$

$$H_{a}:\exists i\in \left\{1,\ldots ,I\right\}:MD\left(\vec{C_{\alpha ,i,j}}\right)>\varphi_{\alpha ,j}\;\&\;MD\left(\vec{C_{\beta_{2},j}}\right)>\varphi_{\beta_{2}}$$

Since the Mahalanobis projection has two dimensions (i.e., for zero and nonzero parts), we compare the Mahalanobis distance to the Chi-Square distribution with 2 degrees of freedom to identify unusually high contributed information values (De Maesschalck et al., 2000). We provide **Algorithm 2** below which performs the hypothesis test at each iteration of the greedy search algorithm (termed as NPEM : BV). A modified version which performs the hypothesis test after the greedy search algorithm has completed is provided in **Supplementary File** as **Algorithm 2**′ (NPEM : BVS). The algorithm follows the same logic as the univariate case, except that we evaluate the contributed information twice, once for the presence-absence data and once for nonzero counts data, with the most informative feature being decided by the largest Mahalanobis distance. The details for obtaining the final p-values are the same as for the univariate test approach.

**Algorithm 2:** Non-Parametric Entropy Mediation: Bivariate Test (NPEM:BV).

Input: ***A*** = {*A*_1_,*A*_2_,…,*A_K_*}: Set of explanatory variables; *B*: Response variable
1. Initialize an empty set ***W***.
2. For each mediator, decompose into presence-absence and nonzero count (*Z*,*M*′)
3. Evaluate Contributed Information for both parts (e.g. $\vec{C_{i}}=\left\{C_{Z}=C\left(A_{i},Z,W\right),C_{M^{\prime }}=C\left(A_{i},M^{\prime },W\right)\right\}$) for each *A_i_* which is not in ***W***.
4. Evaluate the Mahalanobis distance for each vector of contributed information scores $\vec{C_{i}}$ .
5. Move variable *A_k_* into set ***W***.
6. Calculate the Chi-Square Test Statistic: $\chi^{2}=MD(\vec{C_{\left(1\right)}})$
7. If the p-value is below a threshold (e.g., 0.05), move the variable *A*_(1)_ corresponding to the largest Mahalanobis distance $MD(\vec{C_{\left(1\right)}})$ into set ***W***.
8. Repeat steps 3 through 7 until a specified threshold is reached (e.g. 0.05) or until two or fewer variables remain.
9. For the variables which do not belong to W, assign the p-value to be 1.
10. For each response variable, apply FDR correction to the p-values of all explanatory variables.

### Data

#### Simulation Studies

To evaluate the performance of NPEM, we compare our method to existing methods, a nonparametric permutation test, MedTest (Boca et al., 2014), and a method developed to handle SNP counts data, Integrative Genome Wide Association Study, iGWAS (Huang et al., 2015). We simulate biological data for a dichotomous clinical outcome (e.g., healthy or diseased) under various model settings. Gene expression data was simulated for 300 genes using a normal distribution. The first 150 were generated using a standard deviation of 0.5, and the second half with 2.0. Taxon counts were generated using a negative binomial distribution with excess zeros added, with the probability of excess zeros weighted by the log ratio of abundance to population mean (see the **Supplementary File**). The relationships between variables are presented in **Table 1** below.

**Table 1:** Existence of relationships for combinations of gene and taxon indices. True mediation effects require *γ′* (total effect), *α*, and *β_2_* relationships. Here taxa 1–10 are the true mediators for genes 1–20, and taxa 151–160 are the mediators for genes 151–170. The rest taxa are not mediators.

	Low expression σ = 0.5		High expression σ = 2	
	Genes 1–20	Genes 21–150	Genes 151–170	Genes 171–300
Taxa 1–10	*γ′,α,β_2_*	*β_2_*	*γ,β_2_*	*β_2_*
Taxa 11–20	*γ′,α*		*γ′*	
Taxa 21–30	*γ′,β_2_*	*β_2_*	*γ′,β_2_*	*β_2_*
Taxa 31–150	*γ′*		*γ′*	
Taxa 151–160	*γ′,β_2_*	*β_2_*	*γ′,α,β_2_*	*β_2_*
Taxa 161–170	*γ′*		*γ′,α*	
Taxa 171–180	*γ′,β*_2_	*β_2_*	*γ′,β_2_*	*β_2_*
Taxa 181–300	*γ′*		*γ′*	

Three separate simulation studies are performed to examine the behaviour of NPEM under different scenario settings:

- The first study investigates the performance of different models with various sample size (40 and 80 per group) and excess zero probabilities at a high level (80%) or low level (50%) for a total of four data scenarios. The signal strength is fixed at 50%, which is defined as follows:
  $$\text{signal stregth}=\frac{\delta }{\sigma }$$
  where *δ* represents the average difference between healthy and diseased groups and *σ* represents the standard deviation of the noise.
- In practical studies, the signal strength is unlikely to be large for each taxon. We investigate how these methods perform as signal strength decreases by varying signal strength between 50% and 10%. In this simulation, we also vary the excess zero proportions between high (80%) and low (50%), with a fixed sample size 40 per group.
- For further investigation, we observe the effects by increasing the over-dispersion of taxon counts. The over-dispersed counts are modeled using a negative binomial model with the dispersion parameter as follows:
  $$\kappa =\frac{c}{\sqrt{\lambda +1}}.$$
  where *λ* represents the mean count and the constant *c* is set to 1000 for high dispersion and 100 for low dispersion. We fix the sample size to be 40 per group and the excess zero proportion at high (80%) to capture the worse-case. Signal strength ranges from 10% to 50% as in simulation (ii).

For each scenario a total of 20 data sets are generated and evaluated. The results of the simulation studies are presented in *Results*.

#### Pouchitis Data

Pouchitis, inflammation of a post-operation ileal pouch, affects almost half of all ileal pouch-anal anastomosis recipients, with up to 20% of these patients developing chronic pouchitis. We apply NPEM to pouchitis patient data from Morgan et al. (2015), including host gene expression, microbial abundance, and clinical diagnosis, to investigate the relationship of the host gene expression and microbiome. While extensive research has shown host gene expression and the microbiome can influence pouchitis, the causal mechanisms and interactions are not studied well and the authors only found weak association between host gene expression and the microbiome’s effects on the clinical diagnosis.

The clinical data includes samples from 219 patients with information about body location, inflammatory score, antibiotic use, and clinical diagnosis of “No Pouchitis”, “Acute Pouchitis”, “Chronic Pouchitis”, “Crohn’s Disease-Like”, and “Familial Adenomatous Polyposis”. For comparison purposes, we have limited our study to patients with either “No Pouchitis” or “Acute Pouchitis” diagnoses, and no prescribed antibiotics given. This results in an effective sample size of 101 patients. Gene expression data contains 33,297 genes. Transcripts were filtered to remove genes with no annotation, and a log-2 fold change with a conservative cut-off of 0.15 was used to trim the gene set. After filtering, 1103 genes remained. High throughput next-generation sequencing microbiome abundance data recorded 293 operational taxonomic units (OTUs) at the genus level. OTUs that were absent in over 90% of patients were removed, resulting in 103 OTUs.

## Results

### Simulation Study Results

**Figure 3:**
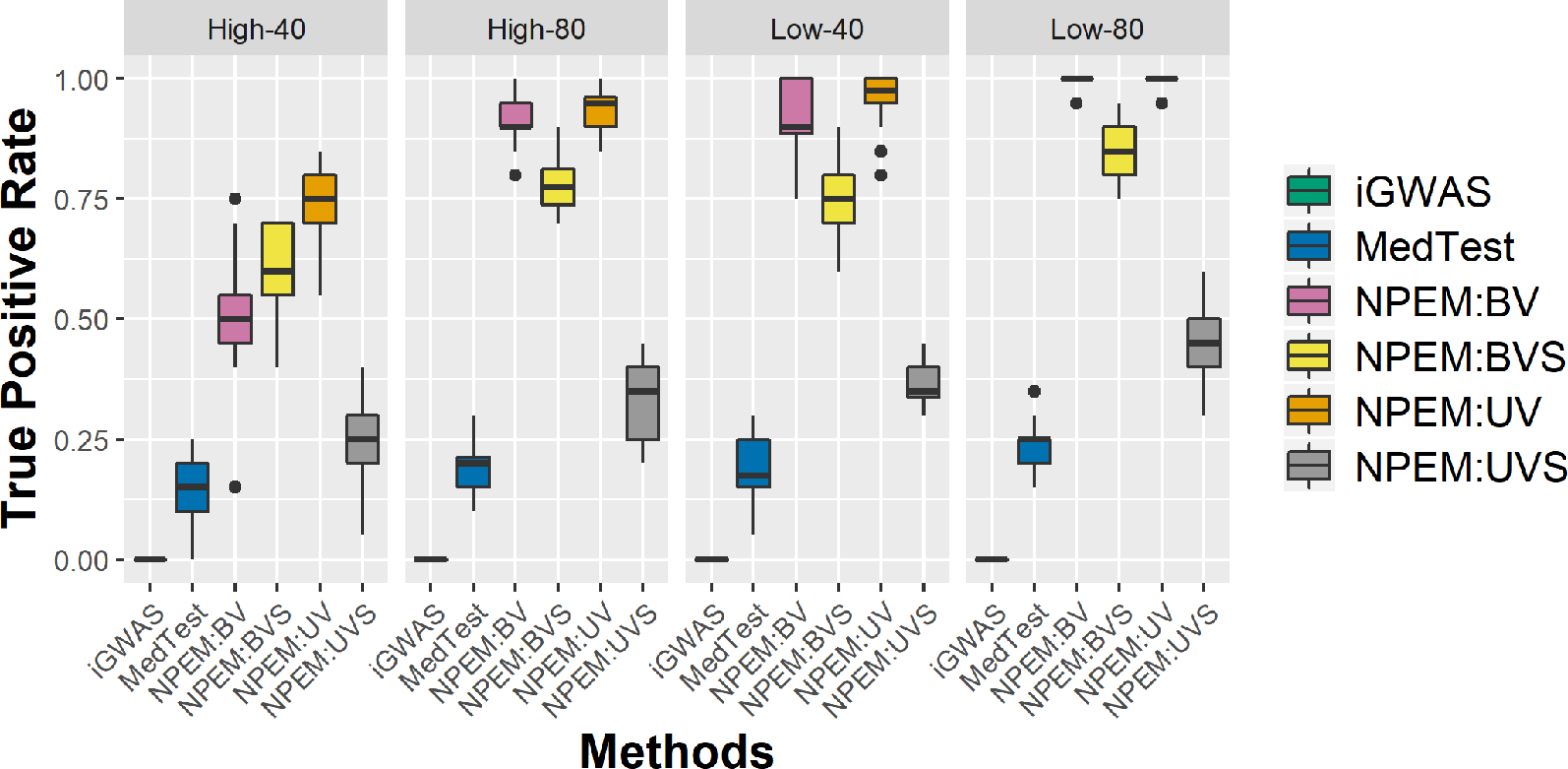
Power plots for simulation studies (i). Sample sizes (40 and 80 per group) and proportions of zero (Low vs. High), with a fixed high signal strength.

**Figure 4:**
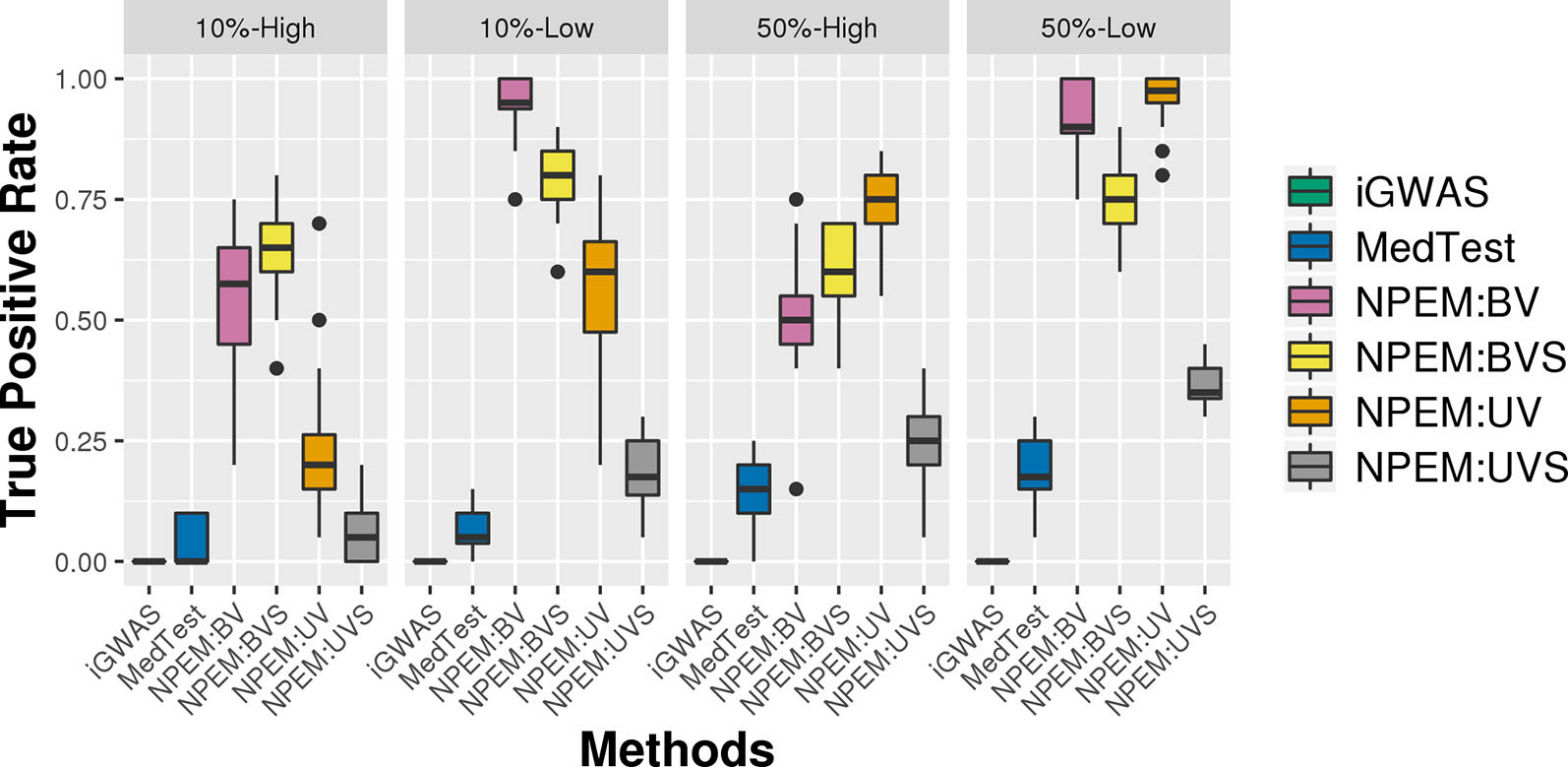
Power plots for simulation studies (ii). Signal strength (50% and 10%) and proportions of zeros (Low vs. High), with a fixed sample size.

With a false positve rate of 5%, NPEM algorithms have higher power than MedTest, while iGWAS fails to discover any significant mediators (**Figure 3**). From the study (i) where the signal strength is high, we find that the UV version of the univariate approach consistently performs the best and the UVS does not perform as well as other NPEM algorithms. Particularly, for a high proportion of zeros and small sample size the UV surpasses the others. As the signal size decreases from 50% to 10% (**Figure 4**), the performance of this univariate test decreases, regardless of the levels of proportion of zeros. However, the bivariate approach maintains better performance. In particular, the single test (BVS) of the bivariate approach is the most consistent and has the highest power when the proportion of zeros in the dataset is high; for a lower proportion of zeros the BV approach is recommended.

**Figure 5:**
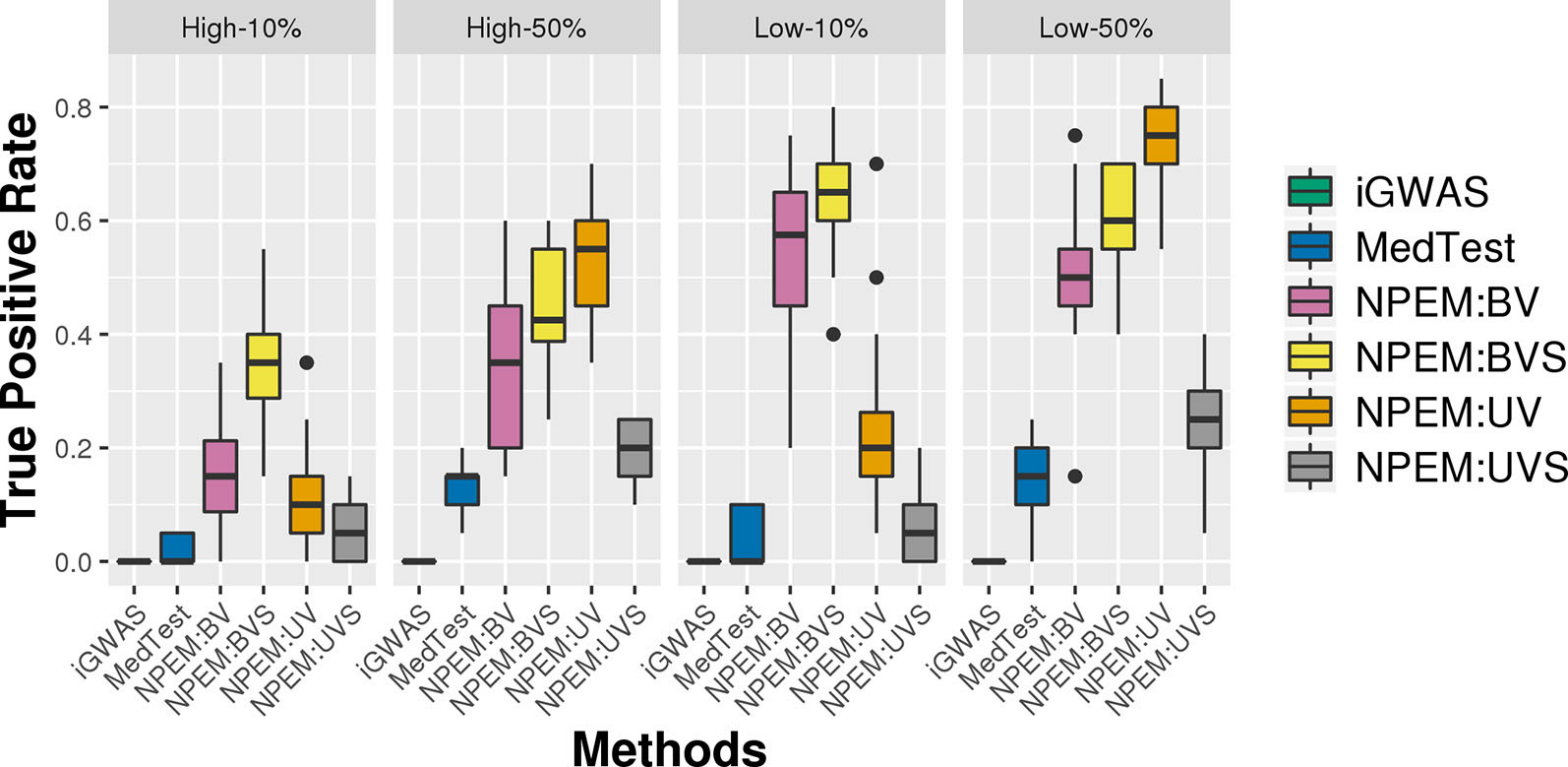
Power plots for simulation studies (iii). Over-dispersion (Low and High) and signal strength (50 and 10%), with a fixed sample size and a fixed proportion of zeros.

For the overdispersion study (i.e., setting iii), the lower the overdispersion, the higher the power (**Figure 5**). The UV approach always outperforms the alternatives when the signal strength is higher, regardless the overdispersion levels; the BVS is always the superior method when the signal size is lower.

**Figure 6:**
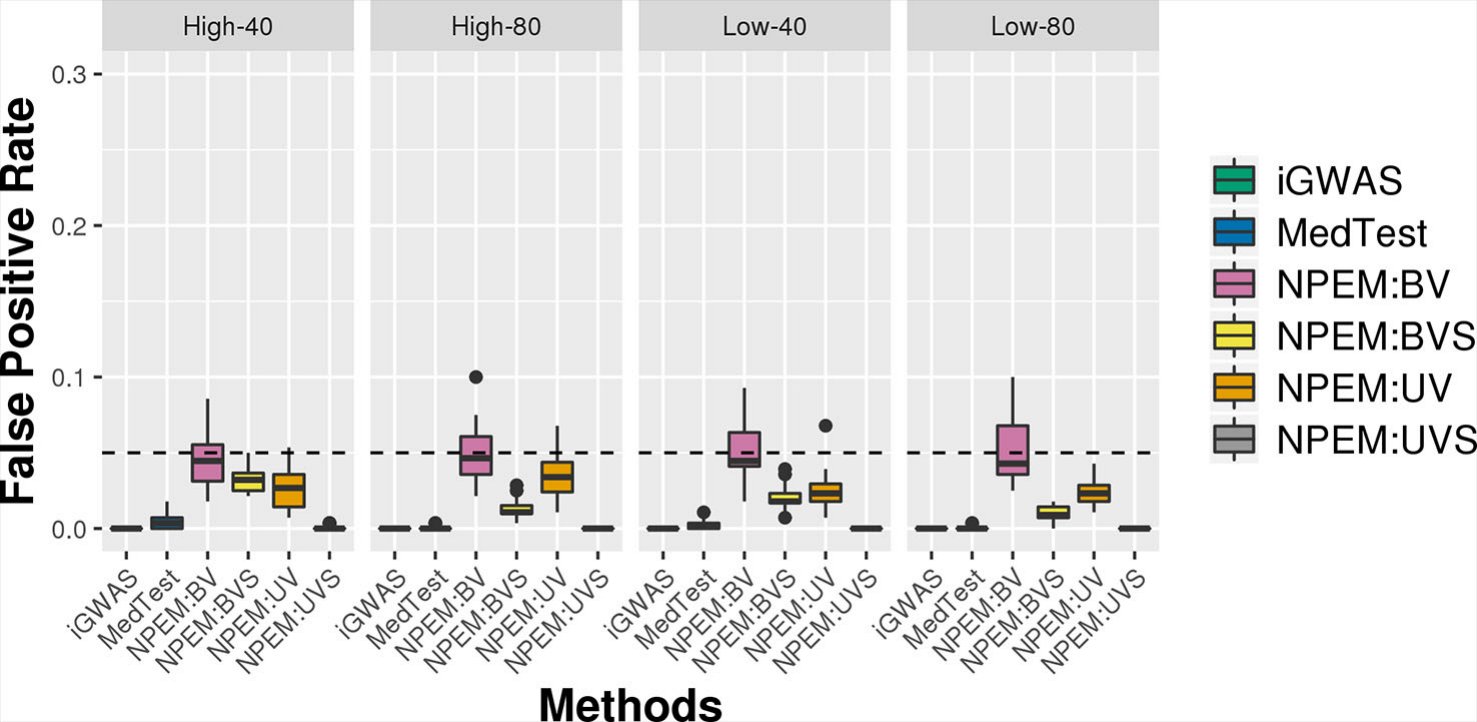
False positive rate plots for simulation studies (i). Sample sizes (40 and 80 per group) and proportions of zeros (Low vs. High), at a fixed signal strength.

For all simulation settings and all methods, the empirical false positive rates are well controlled at pre-specified level. For instance, under simulation setting (i) and using an adjusted p-value cut-off at 0.05, the false positive rates are well controlled (**Figure 6**). The results for settings ii) and iii) are available in the **Supplementary File**.

### Pouchitis Study Results

**Figure 7:**
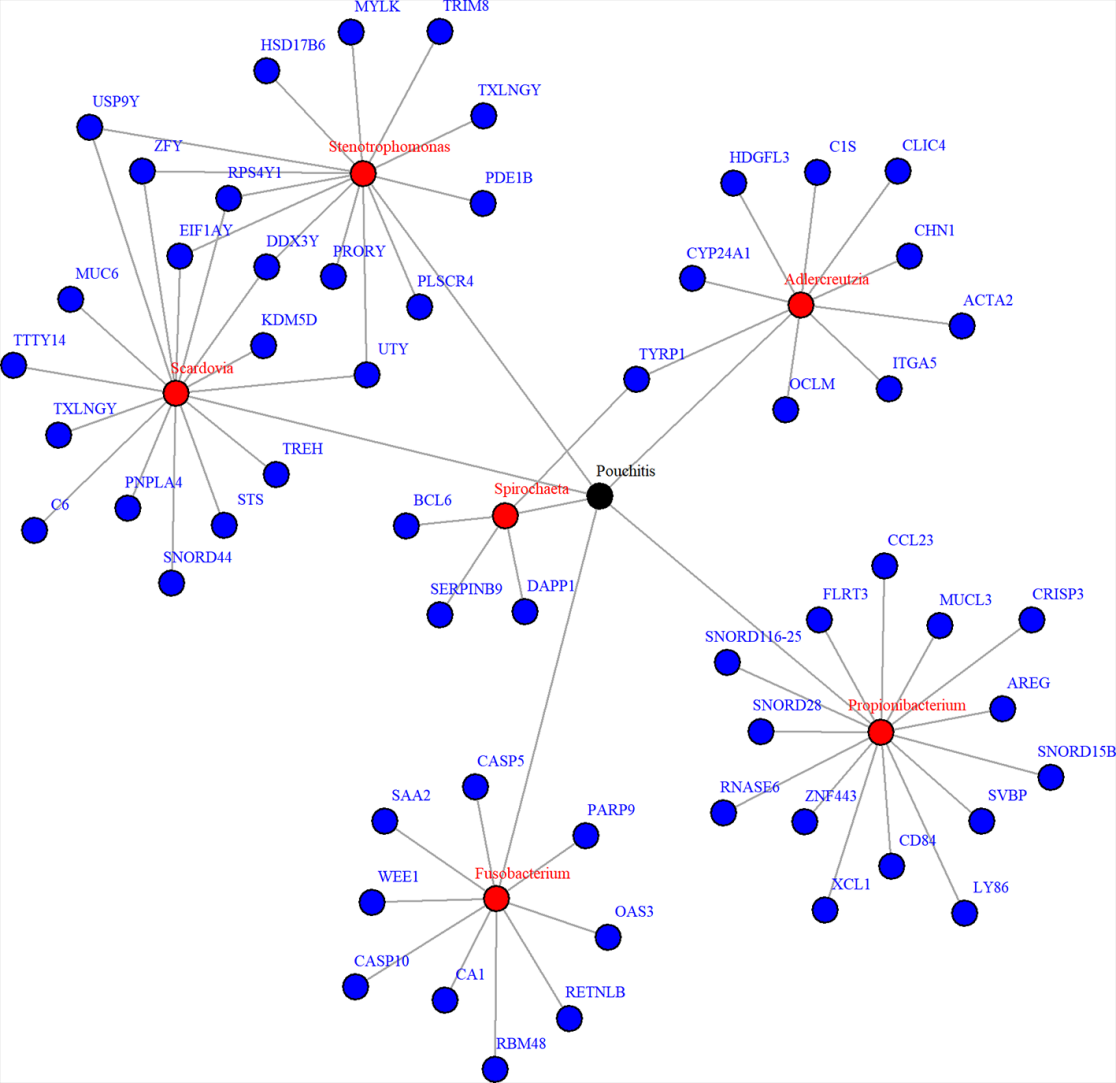
Network plot for significant mediation relationships for detected microbes and associated genes using NPEM : BVS in the Acute Pouchitis study.

Due to zero proportions ranging from 20% to 90%, moderate sample size, and small expected signals in the pouchitis OTU data, we applied the proposed approach BVS on this dataset. Six mediating OTUs were detected at 5% FDR level and the corresponding genera are summarized in **Table 2**. To visualize the relationship between the detected genera and their significantly associated genes, a network plot using significant relationships identified by NPEM : BVS is provided in **Figure 7**.

**Table 2:** Top 6 selected Genera with adjusted P-values from NPEM : BVS algorithm.

Genus	Adjusted p-value
Spirochaeta	4.13E-05
Adlercreutzia	1.96E-04
Propionibacterium	2.15E-04
Scardovia	2.86E-03
Stenotrophomonas	8.34E-03
Fusobacterium	8.91E-03

While research on how bacteria impacts the body is still ongoing, the selected microbial genera are well known to be related to intestinal health. Fusobacterium and stenotrophomonas are well known to be pro-inflammatory (Sasaki and Klapproth, 2012; Shaw et al., 2016), while propionibacterium has recently been found to regulate inflammatory response (Ple et al., 2015; Colliou et al., 2017). Fusobacterium and aldercreutzia are also found to relate to the health of the host mucosal wall (Shaw et al., 2016). Degraded mucosal walls may lead to greater risk of infections due to bacteria growing in the folds of the intestinal wall. Scardovia and spirochaeta have been commonly discovered to be associated with ulcerative and ischemic colitis (Lee et al., 1971; Sasaki and Klapproth, 2012; Xun et al., 2018), two of the primary diseases resulting in ileal pouch-anal anastomosis. Though the exact mechanisms are yet understood, these choices correlate to existing findings and suggest further research is necessary.

When looking at the selected genes, we see a few unique patterns. A number of genes, particularly those related to Scardovia and Stenotropohomonas, are only located on the Y chromosome. Patient gender was not included in the provided metadata, so we were not able to test whether this effect is somehow related to gender or the specific gene. Many selected genes are in the Caspases (CASP) or Small Nucleotide RNA C/D Box (SNORD) groups. CASP genes regulate inflammation response (Scott and Saleh, 2007), which is what we expect. The SNORD gene group regulates expression of other gene groups. In particular, recent research has found correlation between SNORD-116 segments and gut metabolism (Qi et al., 2016). These genes may be a prime candidate for future research.

## Discussion

In this paper, we propose nonparametric entropy models to discover significant mediation structures for microbial mediators. This method is flexible and capable in handling continuous, discrete, and mixed data types for any variable in the model. Though we only discuss continuous and categorical data here, ordinal data may be used in the model by applying a modified Wang-van Ryzen kernel as proposed by Li and Racine (2003) or any other appropriate kernel type. Through simulation studies, we have shown that NPEM outperforms the existing nonparametric test and count-based regression model. In application, our method identifies unique mediation structures undiscovered in the original report relating inflammatory bacteria to host gut health.

The performance of NPEM depends on the data characteristics and selected test statistic. The signal strength in the data is the largest factor separating the performance of the univariate and bivariate options. The bivariate single test (BVS) method is recommended for weak signal size. For the test statistic selection, the poor performance of a singular Grubb’s test is expected; the Grubb’s test is designed to select singular outliers, thus requires sequential selection. Comparison between the bivariate Chi-Square tests is not straightforward since the correlation structure is re-evaluated at each step of the sequential selection algorithm. The proportion of zeros in the data also affects the test selection. When the excess zero proportion is high, a singular test performs stronger than a sequential test. It is important to recognize that the Mahalanobis distance metric does not consider directionality, and unusually low signals may also be selected. A detailed check may be helpful when the noise signals are large.

The alternative causal compositional mediation model, CCMM (Sohn and Li, 2019) was attempted, however, due to the high proportion of zeros and large number of taxonomic units in our experiment, the CCMM algorithm failed to converge. In toy data experiments with no zero counts, CCMM displays higher power in detecting mediating taxa, however it produces much higher false positive rates for associations between host gene expression and taxonomic abundance since the method does not correct for correlation between exposures. The NPEM methods perform much stronger at detecting the correct associations for this particular path α. CCMM is proposed for continuous response, though theoretically a logit link function could handle a binary response.

The performance of our model may be improved through further tuning. The Gaussian kernel is chosen for approximating log-expression density functions for its smoothness and continuous properties. Other kernel types may provide a more accurate fit of the true distribution. Further research is necessary to conclusively decide on the optimal kernel structures for a given dataset. Additionally, the information metrics may be more accurately estimated by implementing leave-one-out cross-validation at the cost of decreased computation speed. However, this research will be the first research to explore the mediation effect from a brand new point of view, an information-based theory.

## Data Availability Statement

16S sequence data for this project was downloaded from Bioproject PRJNA269954 [https://www.ncbi.nlm.nih.gov/bioproject/?term=PRJNA269954]. Microarray data are available from GEO as GSE65270 [https://www.ncbi.nlm.nih.gov/geo/query/acc.cgi?acc=GSE65270]. Metadata are available at [http://huttenhower.sph.harvard.edu/pouchitis2015].

## Author Contributions

LA and KC conceived the study. KC designed the methods and algorithms. LA and ML assisted in tuning and critiquing proposed methods. LA and HJ proposed the real data analysis and KC performed the real data analysis. KC drafted the manuscript and all authors edited it.

## Funding

This work was partially supported by the National Science Foundation [DMS-1222592 to LA]; and the United States Department of Agriculture [ARZT-1360830-H22-138 and ARZT-1361620-H22-149] to L.A.

## Conflict of Interest

The authors declare that the research was conducted in the absence of any commercial or financial relationships that could be construed as a potential conflict of interest.

## References

[B1] Agler R. De Boeck P. (2017). On the Interpretation and Use of Mediation: Multiple Perspectives on Mediation Analysis. Front. In Psychol. 8, 1984. 10.3389/fpsyg.2017.01984 PMC5694788 29187828

[B2] Baron R. M. Kenny D. A. (1986). The moderator-mediator variable distinction in social psychology research: Conceptual, strategic, and statistical considerations. J. Pers. Soc. Psychol. 51, 1173–1182. 10.1037/0022-3514.51.6.1173 3806354

[B3] Benjamini Y. Hochberg Y. (1995). The False Discovery Rate: A Practical and Powerful Approach to Multiple Testing. J. R. Stat. Soc. (Meth.) 57, 289–300. 10.2307/2346101

[B4] Blekhman R. Goodrich J. K. Huan K. Sun Q. Bukowski R. Bell J. T. (2015). Host genetic variation impacts microbiome composition across human body sites. Genome Biol. 16, 1. 10.1186/s13059-015-0759-1 26374288 PMC4570153

[B5] Boca S. M. Sinha R. Cross A. J. Moore S. C. Sampson J. N. (2014). Testing Multiple Biological Mediators Simultaneously. Bionformatics 30, 214–220. 10.1093/bioinformatics/btt633 PMC3892685 24202540

[B6] Bonder M. J. Kurilshikov A. Tigchelaar E. F. Mujagic Z. Imhann F. Vila A. V. (2016). The effect of host genetics on the gut microbiome. Nat. Genet. 48, 1407–1412. 10.1038/ng.3663 27694959

[B7] Colliou N. Ge Y. Sahay B. Gong M. Zadeh M. Owen J. (2017). Commensal Propionibacterium strain UF1 mitigates intestinal inflammation *via* Th17 cell regulation. J. Clin. Invest. 127, 3970–3986. 10.1172/JCI95376 28945202 PMC5663347

[B8] Daniel R. M. De Stalova B. L. Cousens S. N. Vansteelandt S. (2015). Causal Mediation Analysis with Multiple Mediators. Biometrics 71, 1. 10.1111/biom.12248 25351114 PMC4402024

[B9] Davenport E. R. (2017). Tooth Be Told, Genetics Influences Oral Microbiome. Cell Host Microbiome 22, 251–253. 10.1016/j.chom.2017.08.018 28910628

[B10] De Maesschalck R. Jouan-Rimbaud D. Massart D. L. (2000). The Mahalanobis distance. Chemom. Intell. Lab. Syst. 50, 1–18. 10.1016/s0169-7439(99)00047-7

[B11] Galla S. Chakrabory S. Mell B. Vijay-Kumar M. Joe B. (2017). Micorobiotal-Host Interactions and Hypterension. Physiology 32, 224–233. 10.1152/physiol.00003.2017 28404738 PMC6347099

[B12] Grubbs F. E. (1950). Sample criteria for testing outlying observations. Ann. Math. Stat 21, 27–58. 10.1214/aoms/1177729885

[B13] Huang Y. Pan W. (2015). Hypothesis Test of Mediation Effect in Causal Mediation Model with High Dimensional Continuous Mediators. Biometrics 72, 2. 10.1111/biom.12421 26414245

[B14] Huang Y. Liang L. Moffatt M. F. Cookson W. O. C. M. Lin X. (2015). iGWAS: Integrative Genome-Wide Association Studies of Genetic and Genomic Data for Disease Susceptibility Using Mediation Analysis. Genet. Epidemiol. 39, 5. 10.1002/gepi.21905 PMC4544880 25997986

[B15] Kim C. Daniels M. J. Marcus B. H. Roy J. A. (2016). A Framework for Bayesian Nonparametric Inference for Causal Effects of Mediation. Biometrics 73, 2. 10.1111/biom.12575 PMC5288310 27479682

[B16] Kurilshikov A. Wijmenga C. Fu J. Zhernakova A. (2017). Host Genetics and Gut Microbiome: Challenges and Perspectives. Trends In Immunol. 38, 633–647. 10.1016/j.it.2017.06.003 28669638

[B17] Lee F. D. Kraszewski A. Gordon J. Howie J. G. R. McSeveney D. Harland W. A. (1971). Intenstinal spirochaetosis. Gut 12, 126–133. 10.1136/gut.12.2.126 5548558 PMC1411533

[B18] Li Q. Racine J. (2003). Nonparametric estimation of distribution with categorical and continuous data. J. Multivariate Anal. 86, 266–292. 10.1016/S0047-259X(02)00025-8

[B19] Liu J. Lin Y. Lin M. Shunxiang W. Zhang J. (2016). Feature selection based on quality of information. Neurocomputing 255, 11–22. 10.1016/j.neucom.2016.11.001

[B20] MacKinnon D. P. Fairchild A. J. Fritz M. S. (2006). Mediation Analysis. Annu. Rev. Psychol. 58, 593–614. 10.1146/annurev.pscyh.58.110405.085542 PMC2819368 16968208

[B21] Mahalanbois P. C. (1936). On the generalized distance in statistics. Proc. Natl. Inst. Sci. India, 2 49–55.

[B22] Meyer P. E. Schretter C. Bontempi G. (2008). Information-Theoretic Feature Selection in Microarray Data Using Variable Complementarity. IEEE J. Sel. Topics In Signal Process. 2, 261–274. 10.1109/JSTSP.2008.923858

[B23] Morgan X. C. Kabakchiev B. Waldron L. Tyler A. D. Tickle T. L. Milgrom R. (2015). Associations between host gene expression, the mucosal microbiome, and clinical outcome in the pelvic pouch of patients with inflammatory bowel disease. Gegome Biol. 16, 67. 10.1186/s13059-015-0637-x PMC4414286 25887922

[B24] Nguyen T. Q. Webb-Vargas Y. Koning I. M. Stuart E. A. (2016). Causal Mediation Analysis with a Binary Outcome and Multiple Continuous or Ordinal Mediators: Simulations and Application to an Alcohol Intervention. Struct. Equ. Model. 23, 3. 10.1080/10705511.2015.1062730 PMC4855301 27158217

[B25] Ple C. Richoux R. Jardin J. Nurdin M. Briard-Bion V. Parayre S. (2015). Single-strain starter experimental cheese reveals anti-inflammatory effect of Propionibacterium freudenreichii CIRM BIA 129 in TNBS-colitis model. J. Funct. Foods 18, 575–585. 10.1016/j.jff.2015.08.015

[B26] Preacher K. J. (2015). Advances in Mediation Analysis: A Survey and Synthesis of New Developments. Annu. Rev. Psychol. 66, 825–852. 10.1146/annurev-psych-010814-015258 25148853

[B27] Qi Y. Purtell L. Fu M. Lee N. J. Aepler J. Zhang L. (2016). SNORD116 is critical in the regulation of food intake and body weight. Sci. Rep. 6, 1. 10.1038/srep18614 26726071 PMC4698587

[B28] Radovic M. Ghalwash M. Filipovic N. Obradovic Z. (2017). Minimum redundancy maximum relevance feature selection approach for temporal gene expression data. BMC Bioinf. 18, 1. 10.1186/s12859-016-1423-9 PMC5209828 28049413

[B29] Rogers G. B. Wesselingh S. (2016). Precision respiratory medicine and the microbiome. Lancet Respir. Med. 4, 73–82. 10.1016/S2213-2600(15)00476-2 26700443

[B30] Rooks M. G. Garret W. S. (2016). Gut microbiota, metabolites, and host immunity. Immunology 16, 341–352. 10.1038/nri.2016.42 27231050 PMC5541232

[B31] Rostami N. M. Ishaq S. Al Dulaimi D. Zali M. R. Rostami K. (2015). The Role of Infectious Mediators and Gut Microbiome in the Pathogenesis of Celiac Disease. Arch. Iran. Med. 18, 244–249. 015184/AIM.0010 25841946

[B32] Roulston M. S. (1999). Estimating Errors on Measured Entropy and Mutual Information. Phys. D: Nonlinear Phenom. 125, 285–294. 10.1016/S0167-2789(98)00269-3

[B33] Sasaki M. Klapproth J. A. (2012). The Role of Bacteria in the Pathogenesis of Ulcerative Colitis. J. Signal Transduct. 2012, 704953 10.1155/2012/704953 22619714 PMC3348635

[B34] Scott A. M. Saleh M. (2007). The inflammatory caspases: guardians against infections and sepsis. Cell Death Diff. 14, 23–31. 10.1038/sj.cdd.4402026 16977333

[B35] Shannon C. E. (1949). Communication in the Presence of Noise. Proc. IRE 37, 1. 10.1109/JRPROC.1949.232969

[B36] Shaw K. A. Bertha M. Hofmekler T. Chopra P. Vatanen T. Srivatsa A. (2016). Dysbiosis, inflammation, and response to treatment: a longitudinal study of pediatric subjects with newly diagnosed inflammatory bowel disease. Genome Med. 8, 1. 10.1186/s13073-016-0331-y 27412252 PMC4944441

[B37] Silverman B. W. (1986). Density estimation for statistics and data analysis. Density Estimation Stat Data Anal. 10.1201/9781315140919

[B38] Sohn M. Li H. (2019). Compositional Mediation Analysis for Microbiome Studies. Ann. Appl. Stat 13, 661–681. 10.1214/18-AOAS1210

[B39] Taur Y. Parmer E. (2016). Microbiome mediation of infections in the cancer setting. Genome Med. 8, 40. 10.1186/s13073-016-0306-z 27090860 PMC4835935

[B40] Vanderwheele T. J. Vansteelandt S. (2014). Mediation Analysis with Multiple Mediators. Epidemiol. Method 2, 1. 10.1515/em-2012-0010 PMC4287269 25580377

[B41] Xun Z. Zhang Q. Xu T. Chen N. Chen F. (2018). Dysbiosis and Ecotypes of the Salivary Microbiome Associated With Inflammatory Bowel Diseases and the Assistance in Diagnosis of Diseases Using Oral Bacterial Profiles. Front. In Microbiol. 9, 1136. 10.3389/fmicb.2018.01136 PMC5988890 29899737

[B42] Zhang H. Zheng Y. Zhang Z. Gao T. Joyce B. Yoon G. (2016). Estimating and Testing High-dimensional Mediation Effects in Epigenetic Studies. Bioinformatics 32, 3150–3154. 10.1093/bioinformatics/btw351 27357171 PMC5048064

