# Supplementary material for: An Information-Based Approach for Mediation Analysis on High-Dimensional Metagenomic Data
Source: Front Genet. 2020 Mar 13;11:148. doi: 10.3389/fgene.2020.00148 (PMC7083016; doi:10.3389/fgene.2020.00148)
Supplement: Supplementary file 1 [file DataSheet_1.docx]

*Supplementary Materials*

# Alternative Algorithms – single test

The details of single test algorithms for two procedures (Univariate vs. Bivariate), are listed as following.

**Algorithm 1’: Non-Parametric Entropy Mediation: Univariate – Single Test (NPEM:UVS)**

Input: ***A***$=\{A_{1},A_{2},\ldots,A_{K}\}$*:* Set of explanatory variables; B: Response variable

1. Initialize an empty set **W**.

2. Evaluate Contributed Information $C_{i}=C(A_{i},B,\boldsymbol{W})$.

3. Solve $k=argmax_{i}(C_{i})$.

4. Move variable $A_{k}$ into set ***W***.

5. Repeat steps 2 through 4 until all variables are in set ***W***.

6. Solve $k=argmax_{i}(C_{i})$.

7. For the largest Contributed Information, calculate Grubb’s Test Statistic: $G=\frac{C_{k}-\bar{C}}{sd(\boldsymbol{C})}$, where $\boldsymbol{C}$is the vector of the $C_{i}$ values

8. Perform significance test with the distribution $t_{df-2}$ to obtain the p-value $v_{k}$. *df* is the length of $\boldsymbol{C}$.

9. Remove $C_{k}$.

10. Repeat steps 6 through 9 until a specified p-value is reached (e.g. p=0.5) or until 2 or fewer variables remain.

11. Set remaining p-values to 1.

12. Apply FDR correction on p-values.

**Algorithm 2’: Non-Parametric Entropy Mediation: Bivariate – Single Test (NPEM:BVS)**

Input: ***A***$=\{A_{1},A_{2},\ldots,A_{K}\}$*:*: Set of explanatory variables; B: Response variable

1. Initialize an empty set ***W***.

2. For each mediator, decompose into presence-absence and nonzero count $(Z,M^{'})$

3. Evaluate Contributed Information Metrics $C_{i}=\{C_{Z}=C(A_{i},Z,\boldsymbol{W}),C_{M^{'}}=C(A_{i},M',\boldsymbol{W})\}$

4. Solve $k=argmax_{i}(MD(C_{i}))$.

5. Move variable $A_{k}$ into set ***W***.

6. Repeat steps 3 through 5 until 2 or less variables remain.

7. Calculate Chi-Square test statistics: $G_{i}=MD(C_{i})$ and perform significance test with the distribution $\chi_{2}^{2}$ to obtain p-value

8. Apply FDR correction on p-values

# Data Simulation Formulae

## Gene Expression Data

Log gene expression data was generated for 300 genes using a linear model as follows.

$X_{i,k}=\bar{\mu_{i}}+\delta_{i}^{X}I_{k}+\varepsilon_{i,j}$

$\bar{\mu_{i}}=U(6,8)$

$\sigma_{i}=\left\{ \begin{matrix} 0.5 & i\leq150 \\ 2 & i>150 \end{matrix} \right\}$

$I_{k}$ is sampled from $\{0,1\}$

$\delta_{i}^{X}=\pm\left( \sigma_{i} \right)*signal strength$, for significant genes in the gamma model (sign is sampled randomly), 0 otherwise

The distribution of $\varepsilon_{i}$ was generated using a Gaussian copula with correlation structure $\Sigma_{X}$ and marginal distributions $\varepsilon_{i}^{*}\sim N(0,\sigma_{i})$.

$\Sigma_{X}=\left\{ \begin{matrix} 1 & l=m \\ \omega_{l,m} & l\neq m \end{matrix} \right\}$

20 combinations of significant genes in either the gamma or alpha model were randomly sampled, for these combinations, $\omega_{l,m}$ is sampled from $\{0.2,0.5,0.8\}$. $\omega_{l,m}=0$ otherwise.

$\varepsilon_{i,j}$ was sampled form the resulting distribution.

## Metagenomic Data

Raw taxon counts were generated for 300 taxa using an ecological negative binomial model (parameters are size & mean) as follows.

$\log\left( \lambda_{j,k} \right)=\bar{\eta_{j}}+\delta_{j}^{\lambda}I_{k}+\boldsymbol{X}\alpha_{j}^{\lambda}$

$\bar{\eta_{j}}\boldsymbol{=}4$

$\alpha_{j}^{\lambda}=[\begin{matrix} \begin{matrix} \alpha_{1,j}^{\lambda} & \alpha_{2,j}^{\lambda} \end{matrix} & \begin{matrix} \ldots& \alpha_{I,j}^{\lambda} \end{matrix} \end{matrix}]$

$\alpha_{1,j}^{\lambda}$ is sampled from $\{\pm0.8,\pm1.2\}$ for significant genes in the alpha model (sign is sampled randomly), 0 otherwise

$\delta_{i}^{\lambda}=\pm\left( 1 \right)*signal strength$, for significant taxa in the beta model (sign is product $sign\left( \delta_{i}^{X} \right)\cdot sign(\alpha_{i,j}^{\lambda})$), 0 otherwise

$$\kappa_{j,k}=\frac{c}{\sqrt{\lambda_{j,k}+1}}$$

The distribution of ${M'}_{j,k}$ was generated using a Gaussian copula with correlation structure $\Sigma_{M}$ and marginal distributions $M_{j,k}^{*}\sim NB(\kappa_{j,k},\lambda_{j,k})$.

$\Sigma_{M}=\left\{ \begin{matrix} 1 & l=m \\ \nu_{l,m} & l\neq m \end{matrix} \right\}$

20 combinations of significant genes were randomly sampled, for these combinations, $\nu_{l,m}$ is sampled from $\{0.2,0.5,0.8\}$. $\nu_{l,m}=0$ otherwise.

The raw counts ${M'}_{j,k}$ were sampled form the resulting distribution.

# Additional Simulation Plots


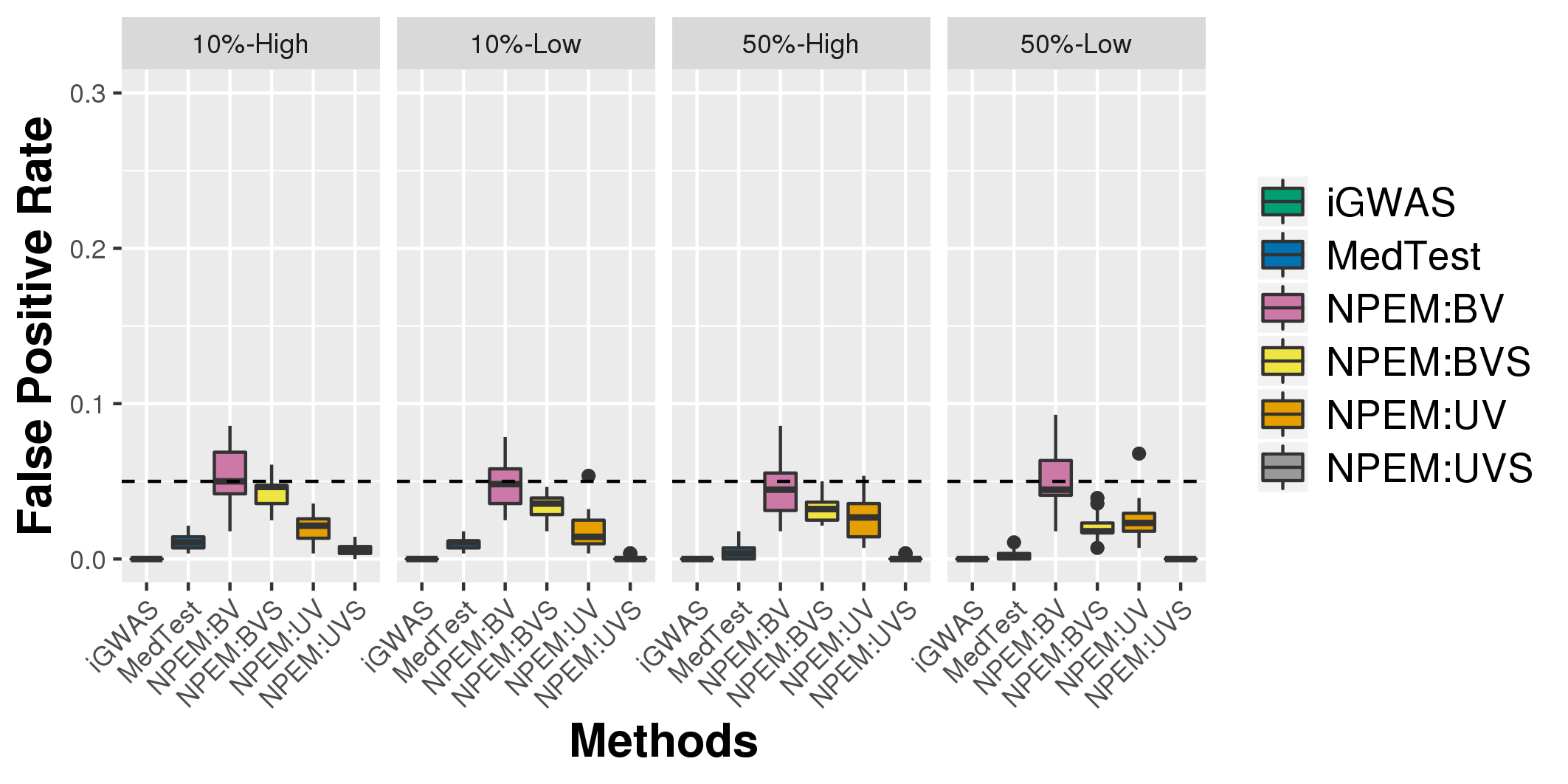


**Figure S1:** False positive rate plots for simulation studies (ii). Signal strength (50% & 10%) and excess zero counts (Low vs. High), for a fixed sample size (40) and over-dispersion.


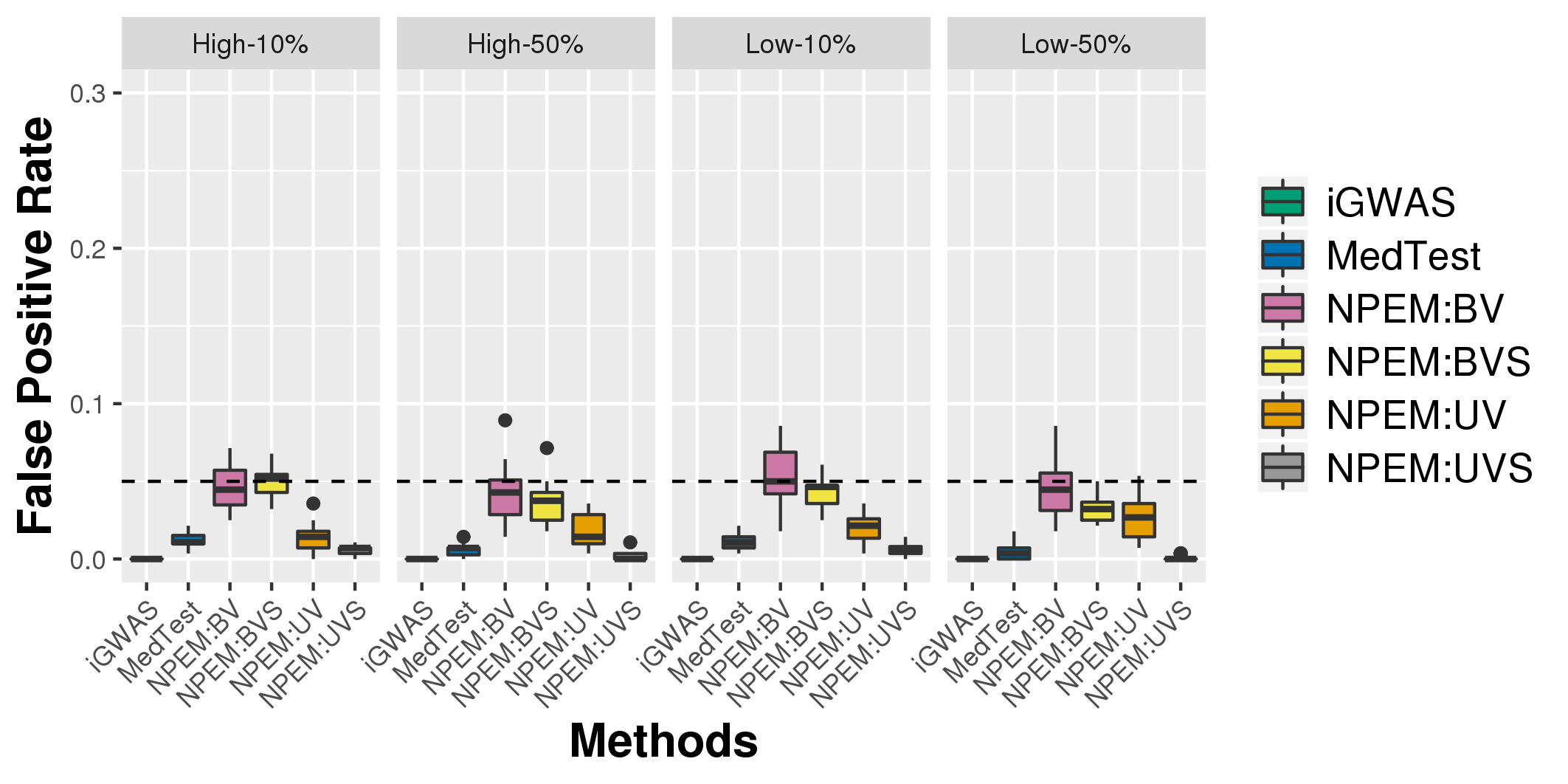


**Figure S2:** False positive rate plots for simulation studies (iii). Over-dispersion (Low & High) and signal strength (50% & 10%), for a fixed sample size (40) and excess zero counts.
